# Prognostic role of intratumoral IL-17A expression by immunohistochemistry in solid tumors: a meta-analysis

**DOI:** 10.18632/oncotarget.18807

**Published:** 2017-06-29

**Authors:** Shimin Wang, Zhi’an Li, Guoming Hu

**Affiliations:** ^1^ Department of Nephrology, Shaoxing People’s Hospital, Shaoxing Hospital of Zhejiang University, 312000, Zhejiang, China; ^2^ Department of Surgical Oncology, Shaoxing Second Hospital, 312000, Zhejiang, China; ^3^ Department of General Surgery, Breast and Thyroid Surgery, Shaoxing People’s Hospital, Shaoxing Hospital of Zhejiang University, 312000, Zhejiang, China

**Keywords:** intratumoral IL-17A overexpression, immunohistochemistry, worse outcome, solid tumor, meta-analysis

## Abstract

IL-17A is an important proinflammatory cytokine which is frequently elevated in tumor microenvironment. However, the role of intratumoral IL-17A in solid tumors remains controversial. Herein, we conducted a meta-analysis to assess the prognostic impact of intratumoral IL-17A in patients with solid tumor. PubMed and EBSCO were searched to identify the studies evaluating the associations between intratumoral IL-17A measured by immunohistochemistry (IHC) and overall survival (OS) and disease-free survival (DFS) in solid tumors. A total of 2972 patients with solid tumor from 21 published studies were incorporated into this meta-analysis. We found that high level of intratumoral IL-17A was significantly associated with worse 3-year, 5-year OS and 1-year, 3-year DFS, but not with 1-year OS or 5-year DFS in solid tumors. In addition, in stratified analyses by cancer types, IL-17A overexpression was significantly associated with worse OS in hepatic carcinoma, but with improved OS in esophageal squamous cell carcinoma (ESCC). Furthermore, high IL-17A expression positively correlated with advanced TNM stage. In conclusion, High expression of intratumoral IL-17A leads to an unfavorable clinical outcome in majority of solid tumors, implicating IL-17A is a valuable biomarker for prognostic prediction of human solid malignances and targeting it may have a potential for effective treatment.

## INTRODUCTION

Chronic inflammation is closely linked to cancer [1, 2], Recent study has demonstrated that cancer-related inflammation promotes tumor development and progression through various mechanisms, including stimulating angiogenesis, promoting cell proliferation and survival, inducing gene mutation and subverting antitumor immune responses [3, 4]. Accumulating evidence has proven that cytokines could promote carcinogenesis by both provoking inflammation [5–7] and eliciting immunosuppression [8, 9].

Interleukin-17A (IL-17A), which initially termed as cytotoxic T-lymphocyte-associated antigen (CTLA-8), is the founding member of IL-17 cytokine family [10, 11]. It is mainly produced by Th17 cells and also by γδ T, natural killer and CD8^+^ T cells [12, 13], relying on STAT3 activation triggered by IL-23 [14]. A large body of evidence suggests that IL-17A is an essential proinflammatory cytokine due to inducing a mass of cytokines and chemokines secretion by distinct cell types, such as mesenchymal cells and myeloid cells, which recruit monocytes and neutrophils into the site of inflammation [15]. Recently, several studies have shown that IL-17A has either a protumor or antitumor role in different cancer models [16]. IL-17A is elevated in several human tumors [17]. In previous studies, high level of IL-17A within tumor was shown to associate with poor survival of several cancers [18–21]; whereas some other studies reported opposite results [22–25]. Therefore, an in-depth assessment is warranted. Moreover, the potential of IL-17A as an effective biomarker in prognostic prediction and targeted therapy is necessary to be explored.

Here, we performed this meta-analysis to test overall survival (OS) and disease-free survival (DFS) as outcomes in patients with solid tumor with known IL-17A levels within tumor evaluated by IHC. The aim of this study was to quantitatively summarize the association between intratumoral IL-17A and clinical outcomes in cancer patients, and thereby provided more evidence on the clinical value of IL-17A as a prognostic biomarker and therapeutic target for solid malignances.

## RESULTS

### Search results and description of studies

**Figure 1:**
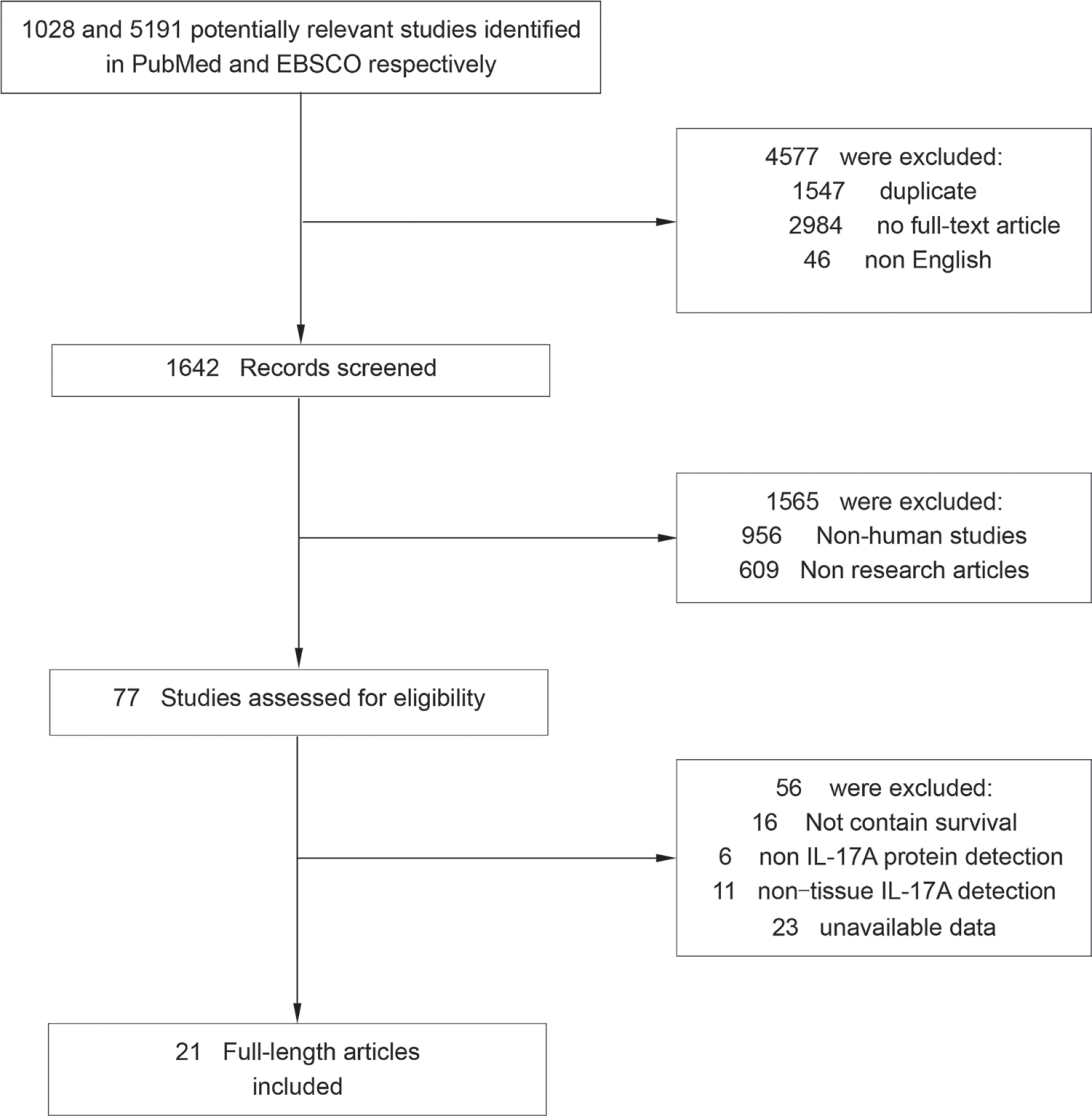
Flow chart diagram of study selection

Literature searches yield 6219 records and the results are shown in Figure 1. 21 studies containing 2972 cancer patients were identified for the assessment of IL-17A expression within tumor [18–38]. All the studies were evaluated by the Newcastle–Ottawa Scale (NOS), and were in accordance with the inclusion criteria and suitable for data consolidation. Characteristics of included studies for OS, DFS and clinicopathological features such as TNM stage, tumor differentiation were shown in Table 1 and Supplementary Table 1 respectively.

**Table 1:** Main characteristics of the included studies

Study	Year	Tumor type	No. of Patients	Male/Female	median age (range) (year)	Cut off for high expression	Tumor stage	median follow-up date (months)	Survival	Quality Score (NOS)
Chen WC, et al. [35]	2013	Breast cancer	207	0/207	51 (23, 78)	positive cells > 90/HPF	I–III	67.2 (7.2, 144)	OS, DFS	8
Chen X, et al. [21]	2010	Non-small cell lung cancer	52	41/11	≥ 60: 40%; < 60: 60%	positive cells > 5%/HPF	I–III	NR	OS, DFS	7
Chen JG, et al. [25]	2011	Gastric cancer	192	129/63	58 (17, 85)	density of positive cells > 2.5/HPF	I–IV	61 (0.3, 81.6)	OS	8
Cui XL, et al. [26]	2013	Glioblastoma	41	18/23	47 (14, 67)	positive cells > 15%	IV	12.9 (4, 24)	OS	7
Gu FM, et al. [28]	2012	Intrahepatic cholangiocarcinoma	123	62/61	55 (18, 78)	positive cells > 111/HPF	I–III	13 (4, 111)	OS	7
Lan CY, et al. [27]	2013	Ovarian cancer	104	0/104	53 (27, 81)	positive cells > 35%/HPF	III–IV	NR	OS	8
Liao R, et al. [18]	2013	Hepatocellular carcinoma	300	253/47	≤ 53: 48%; ≤ 53: 52%	density of positive cells > 51/HPF	I–IV	NR	OS, DFS	7
Liu JK, et al. [29]	2011	Colorectal cancer	52	31/21	≥ 60: 33%; < 60: 67%	positive cells > 5%	III	NR	OS	7
Lv L, et al. [22]	2011	Esophageal squamous cell cancer	181	141/40	≥ 60: 42%; < 60: 58%	density of positive cells > 3.9/HPF	I–IV	NR	OS	8
Zhang JP, et al. [19]	2009	Hepatocellular carcinoma	178	159/19	NR	density of positive cells>7.8cells/0.145mm^2^	I–IV	NR	OS, DFS	7
Zhang GQ, et al. [30]	2012	Non-small cell lung cancer	102	66/36	65 (40, 73)	intensity of staining	I–III	30.2 (24, 59)	OS	7
Lin Y, et al. [23]	2014	Colorectal cancer	78	46/32	≥ 60: 59%; < 60: 41%	score ≥ 3	I–IV	NR	OS	8
Liu XS, et al. [31]	2014	Gastric cancer	112	78/34	60 (33, 89)	density of positive cells/HPF	Tis, I, II, IV	51 (39, 57)	OS	8
Gu FM, et al. [32]	2011	Hepatocellular carcinoma	323	46/277	> 50:48.6%; ≤ 50: 51.4%	density of positive cells	I–III	60 (2, 74)	OS, DFS	7
Li J, et al. [20]	2011	Hepatocellular carcinoma	43	35/8	> 60:18.6% ; ≤ 60:81.4%	density of positive cells > 341/HPF	I–IV	NR	OS, DFS	7
Punt S, et al. [36]	2015	Squamous cervical cancer	109	NR	45 (22, 87)	density of positive cells > 57/0.6mm2	I–IV	112.8 (73.2, 151.2)	OS	7
Zhang Y, et al. [33]	2013	Gallbladder carcinoma	104	63/41	66.13 ± 11.88	positive cells/HPF	I–IV	39 (2, 76)	OS, DFS	7
He SB, et al. [34]	2011	Pancreatic adenocarcinoma	46	31/15	> 60: 63%; ≤ 60: 37%	positive cells >5.60/HPF	I–IV	(5, 48)	OS	6
Wang B, et al. [24]	2013	Esophageal squamous cell cancer	215	160/55	56 (23, 82)	density of positive cells > 10%	I–IV	29.4 (2.2, 156.7)	OS	7
Yu Q, et al. [37]	2014	Cervical cancer	153	NR	≥ 40: 83%; < 40: 17%	NR	II	NR	DFS	6
Tosolini M, et al. [38]	2011	Colorectal cancer	27	NR	NR	density of positive cells > 15/mm^2^	NR	NR	DFS	6

### Meta-analyses

#### Overall survival (OS)

**Figure 2:**
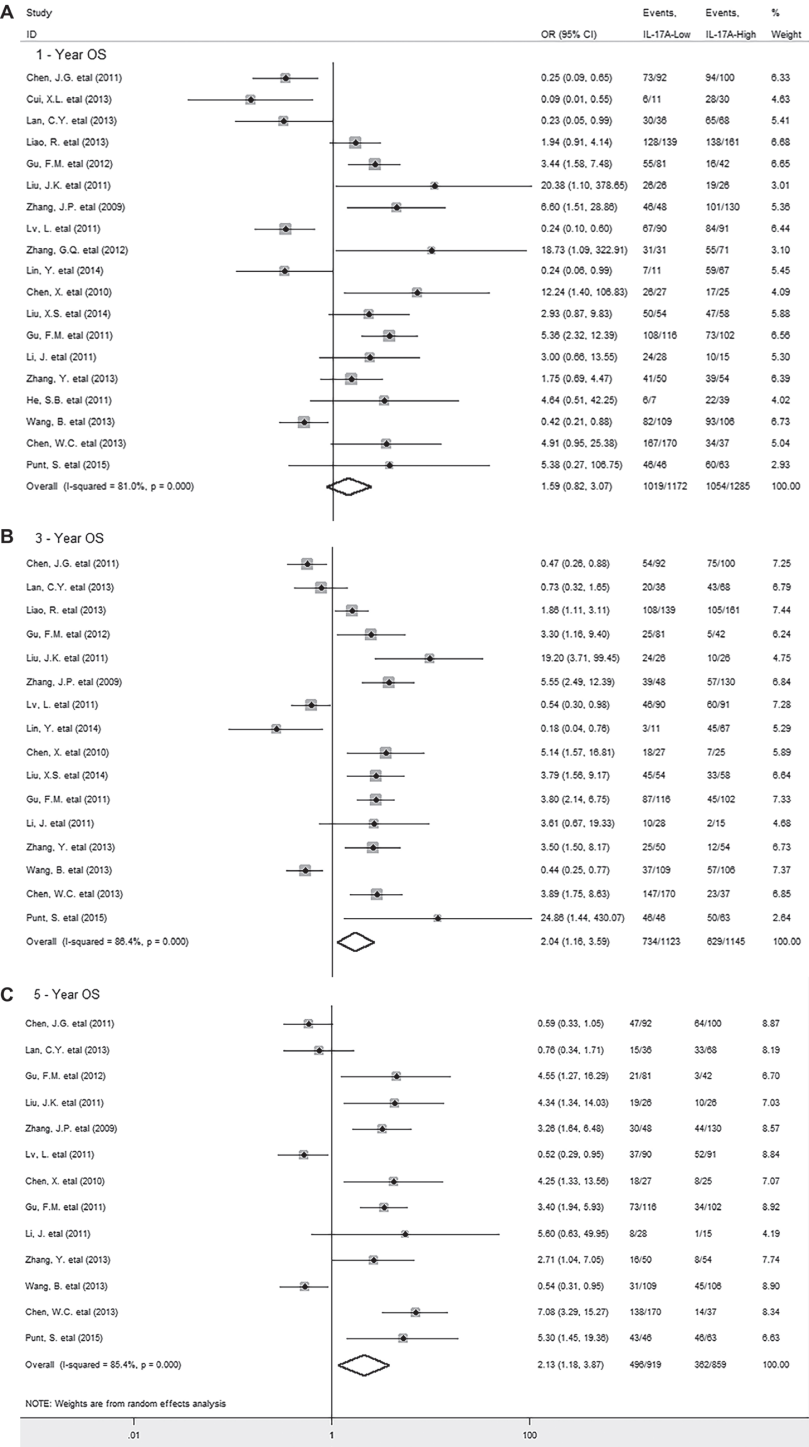
Forest plots describing ORs of the association between intratumoral IL-17A expression and OS at 1-year, 3-year, 5-year

A total of 19 studies with 2562 cancer patients reported the data for OS. The meta-analysis showed that IL-17A overexpression within tumor was not associated with OS in patients with solid tumor (HR = 1.33, 95% CI 0.97 to 1.83, *P* = 0.079) (Supplementary Figure 1), with significant heterogeneity observed among studies (*I*^*2*^ *= 80.2%; P = 0.000*). However, it was important to find that increased level of intratumoral IL-17A was significantly associated with lower 3-year (OR = 2.04, 95% CI 1.16 to 3.59, *P* = 0.014) and 5-year OS rate (OR = 2.13, 95% CI 1.18 to 3.87, *P* = 0.012) in solid tumors (Figure 2B and 2C); whereas there was no significant association between IL-17A and 1-year OS rate (OR = 1.59, 95% CI 0.82 to 3.07, *P* = 0.014) (Figure 2A), with significant heterogeneity observed (*I*^*2*^ *= 81.0%; P = 0.000; I*^*2*^ *= 86.4%; P = 0.000; I*^*2*^ *= 85.4%; P = 0.000* respectively) .

**Figure 3:**
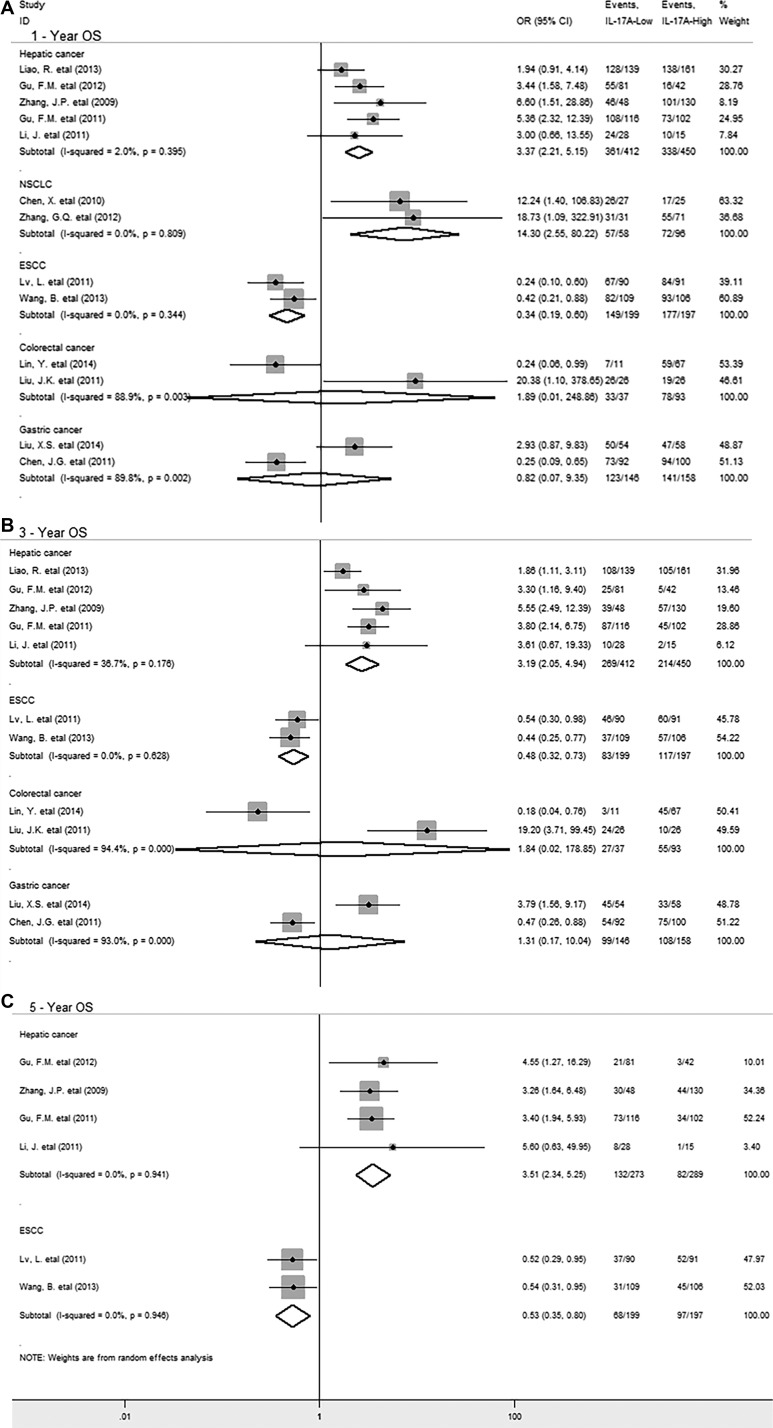
Stratified analyses describing ORs of the association between intratumoral IL-17A expression and OS at 1-year, 3-year, 5-year

In stratified analyses by cancer types, as shown in Figure 3, pooled results showed that IL-17A overexpression was significantly associated with decreased OS at 1-year (OR = 3.37, 95% CI 2.21 to 5.15, *P* = 0.000), 3-year (OR = 3.19, 95% CI 2.05 to 4.94, *P* = 0.000) and 5-year (HR = 3.51, 95% CI 2.34 to 5.25, *P* = 0.000) in hepatic cancer and 1-year OS in non-small cell lung cancer (NSCLC) (OR = 14.30, 95% CI 2.55 to 80.22, *P* = 0.002). However, in esophageal squamous cell carcinoma (ESCC), IL-17A overexpression significantly correlated with improved 1-year (OR = 0.34, 95% CI 0.19 to 0.60, *P* = 0.000), 3-year (OR = 0.48, 95% CI 0.32 to 0.73, *P* = 0.000) and 5-year OS (OR = 0.53, 95% CI 0.35 to 0.80, *P* = 0.002). In addition, there was no significant association between IL-17A and OS at 1-year and 3-year in gastric or colorectal cancer. By the way, there was only one study reporting the relevant data for OS in glioblastoma, breast, ovarian, cervical cancer and gallbladder, pancreatic carcinoma respectively, thus, we couldn’t get a combined result for them.

### Disease-free survival (DFS)

**Figure 4:**
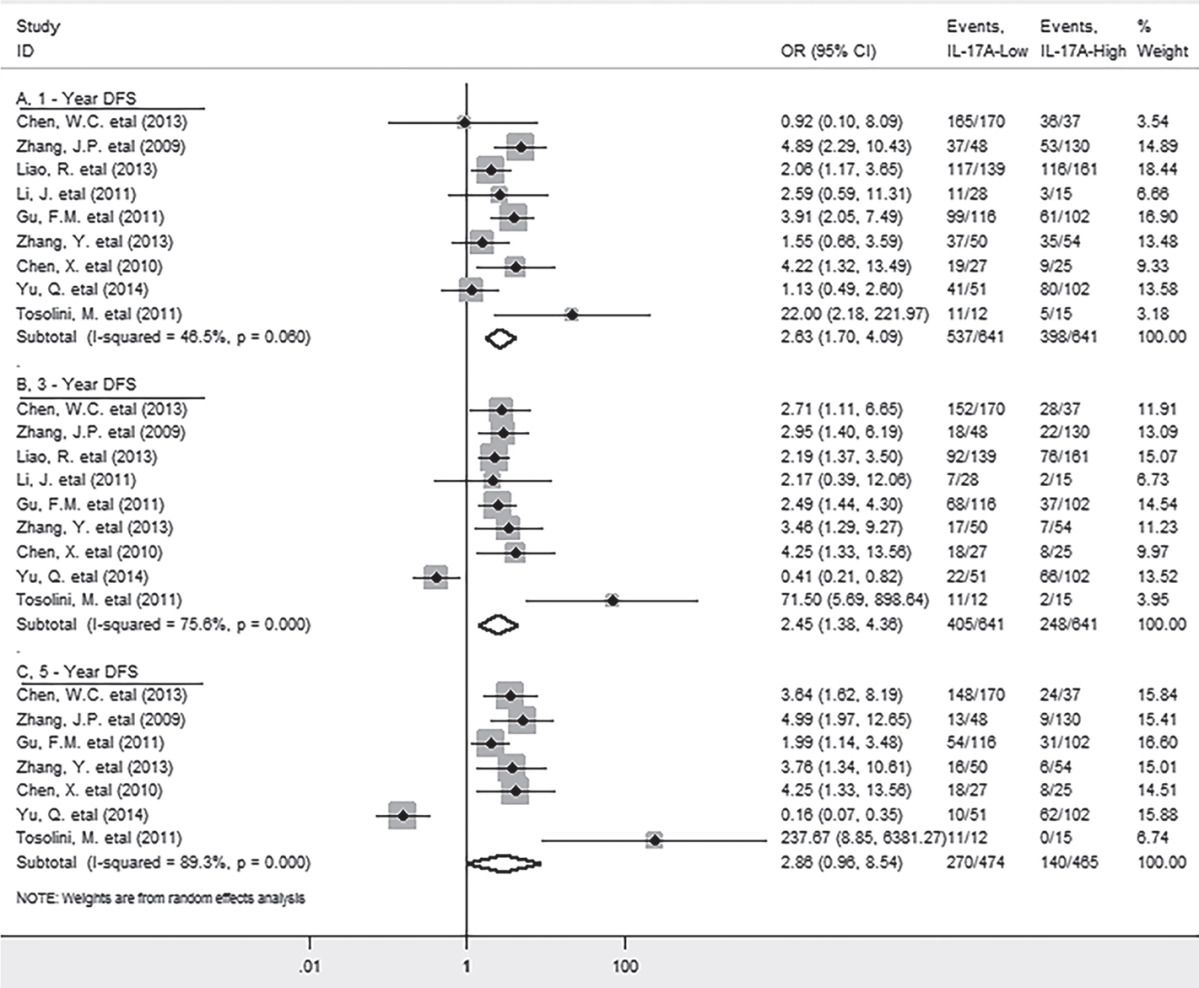
Forest plots describing ORs of the association between intratumoral IL-17A expression and DFS at 1-year, 3-year, 5-year

9 studies reported the data for DFS. Meta-analysis showed that high level of IL-17A within tumor was significantly associated with decreased DFS in solid tumors (HR = 1.70, 95% CI 1.17 to 2.46, *P* = 0.005) (Supplementary Figure 2); while significant heterogeneity was observed (*I*^*2*^ *= 78.9%; P = 0.000*). Specifically, elevated IL-17A was significantly correlated with worse 1-year (OR = 2.63, 95% CI 1.7 to 4.09, *P* = 0.000) and 3-year (OR = 2.45, 95% CI 1.38 to 4.36, *P* = 0.002), but not with 5-year (OR = 2.86, 95% CI 0.96 to 8.54, *P* = 0.059) DFS rate (Figure 4), with significant heterogeneity was observed in the latter two analyses (*I*^*2*^ *= 75.6%; P = 0.000; I*^*2*^ *= 89.3%; P = 0.000* respectively).

**Figure 5:**
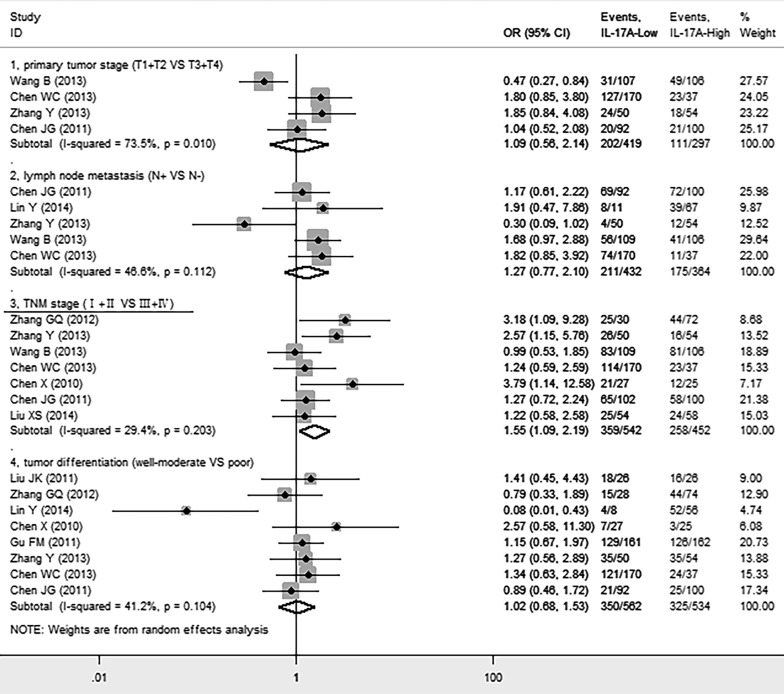
Forest plots indicating ORs of the association between intratumoral IL-17A expression and clinicopathological features

We next investigated whether intratumoral IL-17A was associated with clinicopathological features such as primary tumor (T), lymph node status (N), TNM stage, tumor differentiation of cancer patients. We found that high level of IL-17A was significantly positively correlated with advanced TNM stage (III + IV) (OR = 1.55, 95% CI 1.09 to 2.19, *P* = 0.014), but not with primary tumor (T) (OR = 1.09, 95% CI 0.56 to 2.14, *P* = 0.796), lymph node status (N) (OR = 1.27, 95% CI 0.77 to 2.10, *P* = 0.348), or tumor differentiation (OR = 1.02, 95% CI 0.68 to 1.47, *P* = 1.53) of patients (Figure 5).

### Sensitivity analysis

Sensitivity analyses were used to determine the influence of individual studies on the overall OR. As a result, the plot showed that all the individual studies had no important impact on the results for OS at 1, 3 and 5-year (Supplementary Figure 3).

### Publication bias

Funnel plot and Egger’s test were performed to assess the publication bias of this meta-analysis. No publication bias existed between overexpression of intratumoral IL-17A and OS or DFS in solid tumors (data not shown).

## DISCUSSION

IL-17A shows its bidirectional functions both in pro-tumor and anti-tumor effect. Previous meta-analyses reported that IL-17A wasn’t significantly associated with OS in cancers [39, 40]. However, the studies included in these meta-analyses reported the different sources of IL-17A involving tumor tissue, peripheral blood or peritoneal lavage and different detecting methods such as Flow Cytometry, ELISA, IHC and RT-PCR. Thus, the results were not accurate even wrong when they were from the combinations of all these studies. In this study, we found high level of intratumoral IL-17A was significantly correlated with worse 3-year, 5-year OS and 1-year, 3-year DFS, but not with 1-year OS or 5-year DFS in majority of solid tumors, which differing markedly from the previous studies [39, 40]. Furthermore, IL-17A in tumor tissue positively correlated with TNM stage. Therefore, we believe our study provides meaningful statistical evidence for the prognostic value of intratumoral IL-17A for the first time.

Actually, IL-17A, which is secreted by Th17, γδ T, Tc17 and mast cells in tumor tissue, could promote cancer-elicited inflammation and prevent tumor cells from immune surveillance [17], through subverting T cell mediated anti-tumor immune responses by polarization of myeloid-derived suppressor cells (MDSCs) [41]. In stratified analyses by cancer types, we found the IL-17A overexpression was significantly associated with decreased OS in hepatic carcinoma and NSCLC, but with improved OS in ESCC. The possible explanation might be that the number of intratumoral IL-17A-producing cells positively correlated with infiltrating effector CD8^+^ T and CD57^+^ NK cells in human ESCC as previous reported [42].

Some limitations should be noted from this meta-analysis. First, significant heterogeneity observed across studies cannot be completely accounted. The possible sources of heterogeneity were the inconsistent cut-off values for assessing IL-17A expression and the IL-17A IHC methods taken by different researchers. Therefore, we suggest researchers to use IL-17A mAbs with the same clone number and employ similar cut-off value when they assess IL-17A in the future study. In addition, most of the included studies were performed in China, and studies with negative results or small sample size may not be published, which can cause potential publication bias.

In conclusion, high level of intratumoral IL-17A leads to an unfavorable clinical outcome in majority of solid tumors, implicating IL-17A is a valuable biomarker for prognostic prediction of human solid malignances and targeting it may have a potential for effective treatment.

## MATERIALS AND METHODS

### Search strategy

We searched PubMed and EBSCO for studies assessing the expression of IL-17A in tumor tissue and survival in patients with solid tumor from 1996 to August 2016. The searching keywords were (“Interleukin-17A “ OR “IL-17A” OR “IL17A”) AND (“neoplasms” OR “cancer” OR “tumor” OR “carcinoma”). A total of 1028 and 5191 entries were identified in PubMed and EBSCO respectively.

### Inclusion and exclusion criteria

Inclusion criteria of the study were the measurement of IL-17A by immunohistochemistry (IHC), provided the survival data (OS and /or DFS) and publication in English. Exclusion criteria included studies that evaluated IL-17A with ELISA or Flow Cytometry or RT-PCR, detected IL-17A not in tissues, and studies on animals or in lab.

### Endpoints

OS was recorded as the primary endpoint, while the second endpoint was DFS. Cut-offs of intratumoral IL-17A expression level defined by individual studies classified patients with solid tumor into high- and low- groups.

### Data extraction

Two authors (GM.H. and ZA.L.) independently reviewed and extracted data using predefined data abstraction forms from each eligible studies. Extracted information included first author’s name, publication year, country, type of cancer, number of patients, median age, gender, Tumor, Lymph Node, Metastasis (TNM) stage, tumor differentiation, time of follow-up, technique used to quantify IL-17A, and cut-off value to determine IL-17A positivity. OS, DFS and clinicopathological data were extracted from the text, tables, or Kaplan – Meier curves for both IL-17A-high and -low group.

### Quality assessment

The studies included in the meta-analysis were cohort studies. The quality of each included study was assessed using Newcastle–Ottawa Scale (NOS) (Supplementary Table 2) by two independent authors [43]. (Supplementary Table 3) The studies with 6 scores or more were classified as high quality studies. A consensus NOS score for each item was achieved.

### Statistical analysis

Extracted data were combined into a meta-analysis using STATA 12.0 analysis software (Stata Corporation, College Station, TX, USA). Statistical heterogeneity was assessed using the chi-squared based *Q*-test or the *I*^*2*^ method [44]. Data were combined according to the random-effect model in the presence of heterogeneity [45], otherwise, the fixed-effect model was performed [46]). Sensitivity analysis was employed to assess the influence of each study on the pooled result. Begg’s funnel plot and Egger’s test [47] were calculated to investigate potential publication bias. All *P* values were two-sided and less than 0.05 are considered statistically significant.

## SUPPLEMENTARY MATERIALS FIGURES AND TABLES



## Abbreviations

IL-17A: interleukin-17A
OS: overall survival
DFS: disease-free survival
HR: hazard rations
OR: odds ratios
Cl: confidence interval
IHC: immunohistochemistry
TNM: Tumor, Lymph Node, Metastasis
ELISA: Enzyme-Linked Immunosorbent Assay
RT-PCR: real-time reverse transcription polymerase chain reaction
NR: not reported
ESCC: esophageal squamous cell carcinoma
NSCLC: non-small cell lung cancer
