# Supplementary material for: Prognostic role of intratumoral IL-17A expression by immunohistochemistry in solid tumors: a meta-analysis
Source: Oncotarget. 2017 Jun 29;8(39):66382–91. doi: 10.18632/oncotarget.18807 (PMC5630420; doi:10.18632/oncotarget.18807)
Supplement: Supplementary file 1 [file oncotarget-08-66382-s001.pdf]

Prognostic role of intratumoral IL-17A expression by immunohistochemistry in solid tumors: a meta-analysis

Supplementary Materials

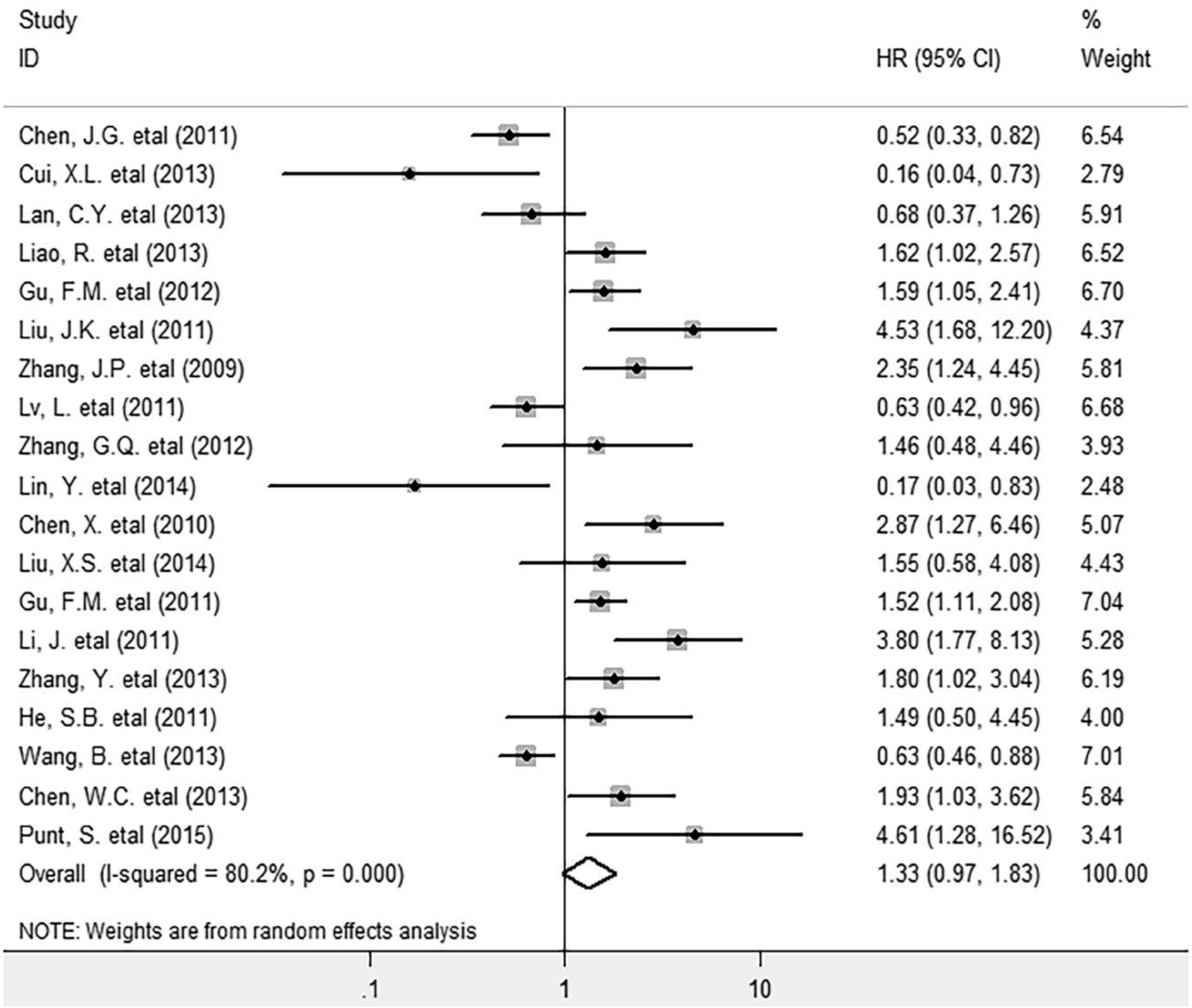

Supplementary Figure 1: Forest plots describing HR of the association between intratumoral IL-17A and OS in solid tumors.

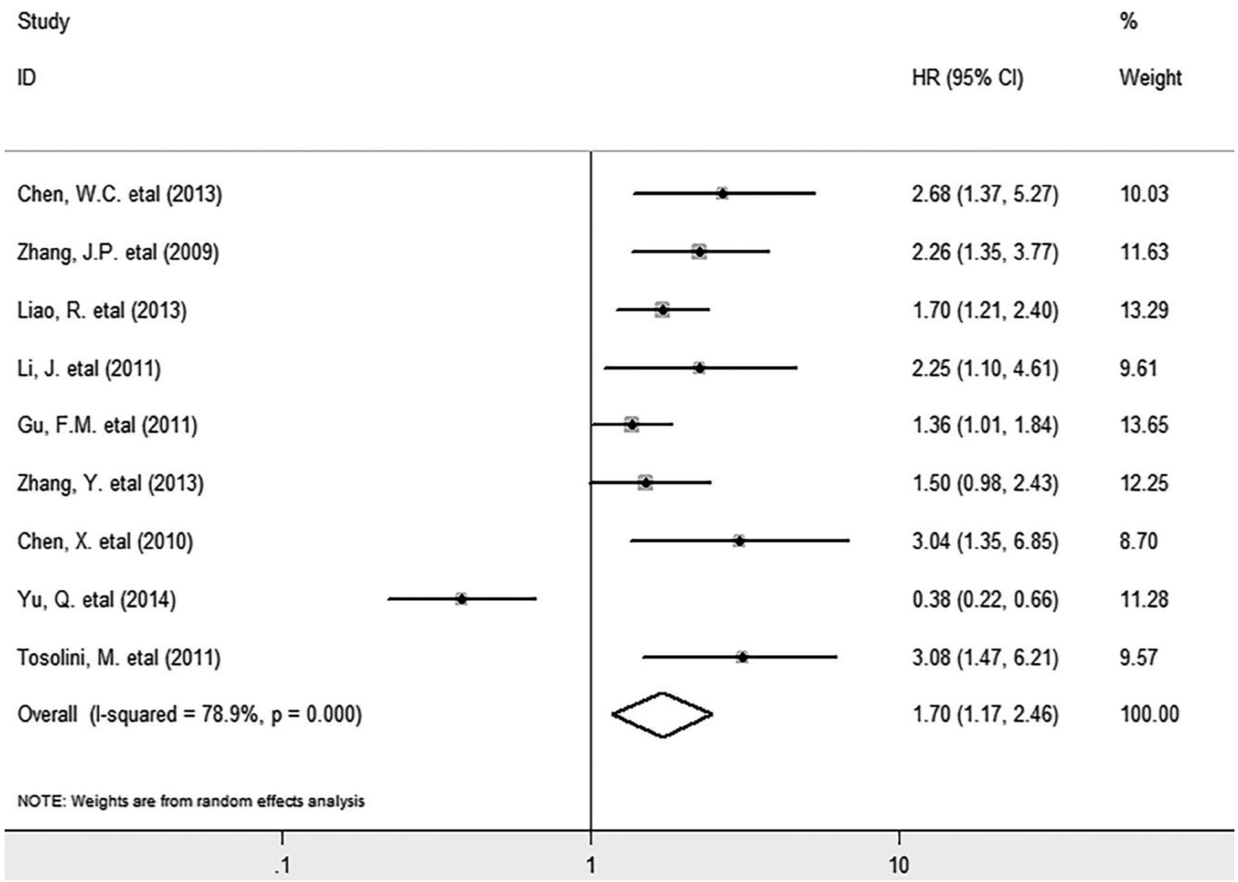

Supplementary Figure 2: Forest plots describing HR of the association between intratumoral IL-17A and DFS in solid tumors.

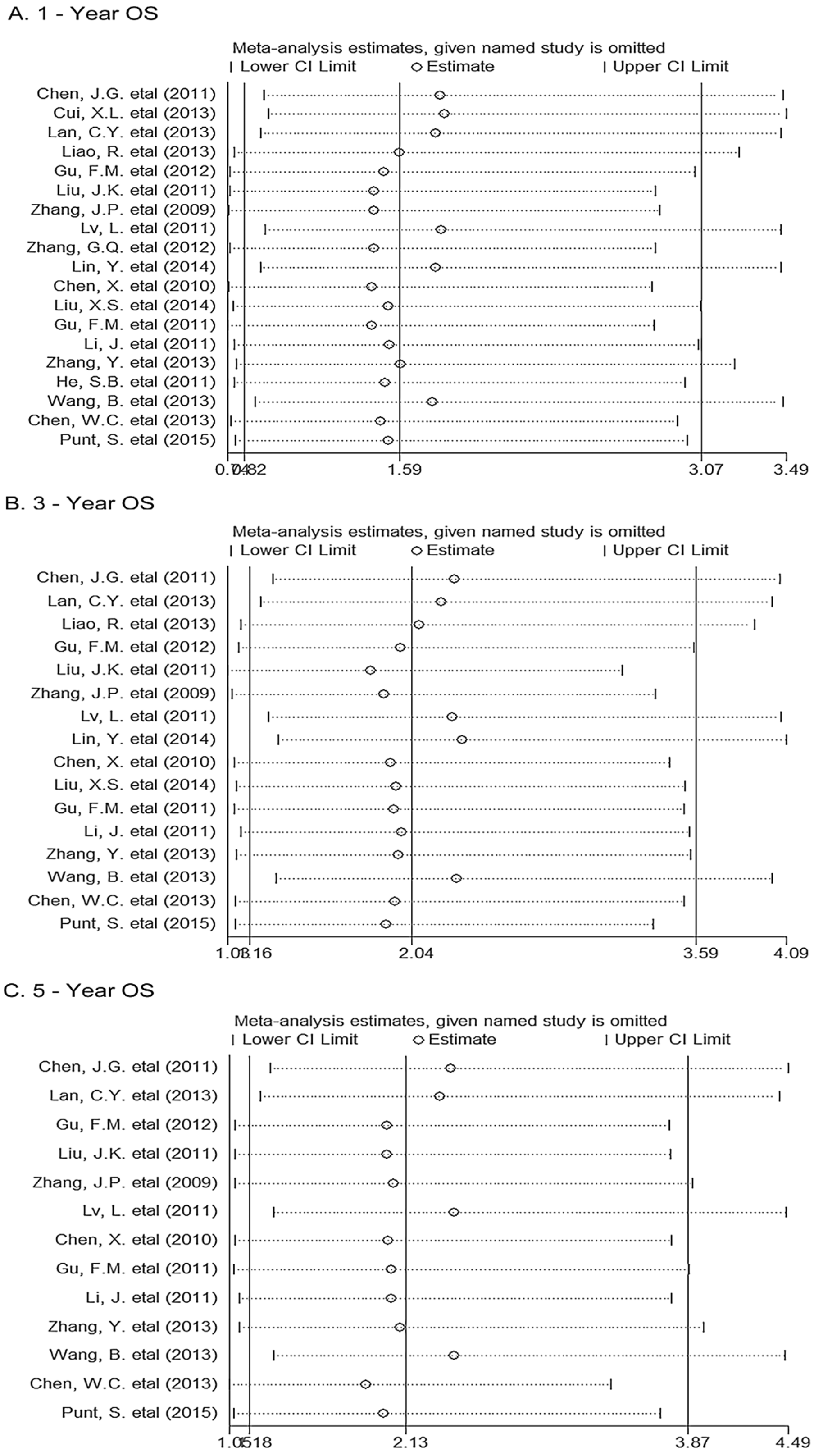

Supplementary Figure 3: Plots describing the influence of individual studies on the OR for OS at 1-year, 3-year, 5-year.

Supplementary Table 1: Characteristics of the included studies for OR analysis of clinicopathological features

Study	Year	Tumor type	No. of Patients	Cut off for high expression	IL-17A High expression (%)	T category (T1+T2 / T3+T4)	N category (Positive / Negative)	M category (M0/M1)	Tumor stage	I + II / III + IV	Tumor Differentiation (Well-moderate/poor)
Chen WC, et al. [35]	2013	Breast cancer	207	positive cells > 90/HPF	37 (17.9)	H: (23/14); L: (127/43)	H: (11/26); L: (74/96)	NR	I–III	H: (23/14); L: (114/56)	H:(24/13); L: (121/49)
Chen X, et al. [21]	2010	Non-small cell lung cancer	52	positive cells >5%/HPF	25 (48.1)	NR	NR	NR	I–III	H: (12/13); L: (21/6)	H:(3/22); L: (7/20)
Chen JG, et al. [25]	2011	Gastric cancer	192	density of positive cells > 2.5/HPF	100 (52.1)	H: (21/79); L: (20/72)	H: (72/28); L: (69/23)	NR	I–IV	H: (58/42); L: (65/37)	H:(25/75); L: (21/71)
Liu JK, et al. [29]	2011	Colorectal cancer	52	positive cells > 5%	26 (50.0)	NR	NR	NR	III	NR	H:(16/10); L: (18/8)
Zhang GQ, et al. [35]	2012	Non-small cell lung cancer	102	intensity of staining	71 (69.6)	NR	NR	NR	I–III	H: (44/28); L: (25/5)	H:(44/30); L: (15/13)
Lin Y, et al. [23]	2014	Colorectal cancer	78	score ≥ 3	67 (85.9)	NR	H: (39/28); L: (8/3)	H: (49/18); L: (6/5)	I–IV	H: (28/39); L: (1/10)	H:(52/4); L: (4/4)
Gu FM, et al. [32]	2011	Hepatocellular carcinoma	323	density of positive cells	162 (50.2)	NR	NR	NR	I–III	NR	H:(126/36);L: (129/32)
Zhang Y, et al. [33]	2013	Gallbladder carcinoma	104	positive cells/ HPF	54 (51.9)	H:(18/36); L:(24/26)	H:(12/42); L:(4/46)	H: (47/7); L: (45/5)	I–IV	H: (16/38); L: (26/24)	H:(35/19); L: (35/15)
Wang B, et al. [24]	2013	Esophageal squamous cell cancer	215	density of positive cells > 10%	106 (49.3)	H:(49/57); L:(31/76)	H:(41/65); L:(56/53)	NR	I–IV	NR	H:(81/25); L: (83/26)

T: primary tumor; N: lymph node; M: metastasis; H: high; L: low; NR: not reported; HPF: high-power field.

Supplementary Table 2: Specific criteria of Newcastle-Ottawa Scale (NOS)

NOTE: Identify 'high' quality choices with a 'star' (one 'star' means 1 score); A maximum of one 'star' for each item within the *'Selection'* and *'Outcome'* categories; maximum of two 'stars' for *'Comparability'*. Using the tool, each study is judged on eight items, categorized into three groups: the 'selection' of the study groups; the 'comparability' of the groups; and the ascertainment of the outcome of interest for cohort studies. Stars awarded for each quality item serve as a quick visual assessment. Stars are awarded such that the highest quality studies are awarded up to nine stars.

1, Selection (4)

(1), Representativeness of the exposed cohort

- a) truly representative of the average ____ (describe) in the community ★
- b) somewhat representative of the average ____ in the community ★
- c) selected group of users eg nurses, volunteers
- d) no description of the derivation of the cohort

(2), Selection of the non exposed cohort

- a) drawn from the same community as the exposed cohort ★
- b) drawn from a different source
- c) no description of the derivation of the non exposed cohort

(3), Ascertainment of exposure to implants

- a) secure record (eg surgical records) ★
- b) structured interview ★
- c) written self report
- d) no description

(4), Demonstration that outcome of interest was not present at start of study

- a) yes ★
- b) no

2, Comparability (1)

(1), Comparability of cohorts on the basis of the design or analysis

- a) study controls for ______ (select the most important factor) ★
- b) study controls for any additional factor (This criteria could be modified to indicate specific control for a second important factor.) ★

3, Outcome (3)

(1), Assessment of outcome

- a) independent blind assessment ★
- b) record linkage ★
- c) self report
- d) no description

(2), Was follow up long enough for outcomes to occur

- a) yes (select an adequate follow up period for outcome of interest) ★
- b) no

(3), Adequacy of follow up of cohorts

- a) complete follow up - all subjects accounted for ★
- b) subjects lost to follow up unlikely to introduce bias - small number lost - > ___ % (select an adequate %) follow up, or description of those lost) ★
- c) follow up rate < ___% (select an adequate %) and no description of those lost
- d) no statement

Supplementary Table 3: Study quality assessment according to the Newcastle–Ottawa Scale (NOS)

Study	Year	Selection				Comparability		Outcome			Quality Score (NOS)
		(1)	(2)	(3)	(4)	(1) - a	(1) - b	(1)	(2)	(3)	
Chen WC, et al. [35]	2013	Yes	Yes	Yes	Yes	No	Yes	Yes	Yes	Yes	8
Chen X, et al. [21]	2010	Yes	Yes	Yes	Yes	No	Yes	Yes	Yes	No	7
Chen JG, et al. [25]	2011	Yes	Yes	Yes	Yes	Yes	No	Yes	Yes	Yes	8
Cui XL, et al. [26]	2013	Yes	Yes	Yes	No	Yes	Yes	Yes	No	Yes	7
Gu FM, et al. [28]	2012	Yes	Yes	Yes	No	Yes	Yes	Yes	No	Yes	7
Lan CY, et al. [27]	2013	Yes	Yes	Yes	Yes	Yes	Yes	Yes	Yes	No	8
Liao R, et al. [18]	2013	Yes	Yes	Yes	Yes	Yes	Yes	Yes	No	No	7
Liu JK, et al. [29]	2011	Yes	Yes	Yes	No	Yes	Yes	Yes	No	Yes	7
Lv L, et al. [22]	2011	Yes	Yes	Yes	Yes	Yes	Yes	Yes	No	Yes	8
Zhang JP, et al. [19]	2009	Yes	Yes	No	Yes	Yes	Yes	Yes	Yes	No	7
Zhang GQ, et al. [30]	2012	Yes	Yes	Yes	No	Yes	No	Yes	Yes	Yes	7
Lin Y, et al. [23]	2014	Yes	Yes	Yes	Yes	Yes	No	Yes	Yes	Yes	8
Liu XS, et al. [31]	2014	Yes	Yes	Yes	Yes	Yes	No	Yes	Yes	Yes	8
Gu FM, et al. [32]	2011	Yes	Yes	Yes	Yes	No	No	Yes	Yes	Yes	7
Li J, et al. [20]	2011	Yes	Yes	Yes	No	Yes	No	Yes	Yes	Yes	7
Punt S, et al. [36]	2015	Yes	Yes	Yes	Yes	No	No	Yes	Yes	Yes	7
Zhang Y, et al. [33]	2013	Yes	Yes	Yes	Yes	No	No	Yes	Yes	Yes	7
He SB, et al. [34]	2011	Yes	Yes	No	Yes	No	Yes	Yes	No	Yes	6
Wang B, et al. [24]	2013	Yes	Yes	No	Yes	Yes	No	Yes	Yes	Yes	7
Yu Q, et al. [37]	2014	Yes	Yes	Yes	No	No	No	Yes	Yes	Yes	6
Tosolini M, et al. [38]	2011	Yes	Yes	Yes	No	No	No	Yes	Yes	Yes	6
